# Editorial Notes

**Published:** 1942

**Authors:** 


### Editorial Notes

**Local Award.** The George Medal has been awarded to Squadron Leader Frank George Mogg, R.A.F., M.S., for his gallantry in fighting his way into the blazing wreckage of a crashed aircraft, and administering morphine to a trapped wireless operator.*

**The M.M.A. Jubilee.** In 1891 the Medical Mission Auxiliary, the M.M.A. for short, was formed to provide for the medical work of the Church Missionary Society. This had formerly been the responsibility of the Society as a whole. The anniversary meetings this year will celebrate the completion of the M.M.A.'s fiftieth year of work. Dr. H. G. Anderson, Medical Superintendent of the Church Missionary Society, has kindly contributed the following account of the origin and progress of the M.M.A. :—

The first recorded worker in the Society to conceive of healing as part of his ministry was the Rev. Abdul Masih, a devout and cultured Indian, one of Henry Martyn's first converts. This was in 1815, but it was not until 1836 that the first English doctor was sent abroad. He went to care for the C.M.S. Mission in New Zealand. He was instructed not only to heal but to train the natives to perform simple medical procedures for their fellows—a shadowing of things to come. Similarly in 1850 a doctor was sent out to Foochow, in China, to begin medical dispensary work there. In 1865, in Kashmir, at the request of an English Government official in India, Dr. William Elmslie opened the first C.M.S. mission hospital. He died there some eight years later, worn out by his work.

During the previous thirty years other doctors had been sent to various parts of the world, notably to West Africa, where health breakdown and mortality amongst our missionaries had been appalling. Their main duty was to act as medical officers to the missionaries themselves. In the next quarter of a century over thirty medical missionaries were commissioned as such. And in the year that the M.M.A. was founded C.M.S. had about as many medical missionaries abroad as had any sister society: a total then of twenty-four doctors. In considering later the enormous increase of the work, it should be remembered that this number has increased only four-fold in the last fifty years. The M.M.A.'s achievements of to-day are in a sense the result not of fifty but of more than a hundred years of experience by trial and error in Africa, India, China, and Mohammedan lands.

As far as available reports show, M.M.A. institutions are caring for more than one out of every ten patients coming to such institutions (of

* By kind permission of the Editor of the *Bristol Evening World.*

whatever creed or country of origin) throughout the world! The M.M.A. was recently, and probably still is, the largest medical mission organization in the world. In the number of beds (nearly six thousand) it compares with the capacity of the twelve famous London voluntary teaching hospitals. To maintain this great medical service a sum equal to only about one-thirtieth of the grants per patient made by our English medical services is available for each C.M.S. patient. More than three-fifths of the total expenditure abroad is met locally; that expenditure in 1939 was about £180,000.

The first published statistics of M.M.A. in 1892 show about 3,900 in-patients and 247,000 out-patient attendances; our latest figures show about 91,000 and 2,460,000 respectively. Moreover, the present annual rate of increase is actually considerably more than our first recorded totals! It is true that trained non-western doctors in our establishment will soon equal in number their missionary colleagues, and that missionary nurses are now only one-tenth of the total trained nursing staff; nevertheless, to maintain such work a fifty per cent. increase in missionary staff has become necessary. The stress and strain even before war came was a heavy burden, and that the work is carried on and grows is due to self-sacrifice and devotion both abroad and at home, which have become a splendid feature of the Church to-day. Sufficient is it to say in illustration that the break-down rate amongst C.M.S. doctors is the highest for our missionaries, and that in certain areas the rate amongst nurses is from three to four times that of other women missionaries.

After all, this Jubilee year celebrates not merely a great medical missionary achievement but those hundreds abroad and thousands at home who have made it possible. It advertises not primarily an imposing list of fifty-two hospitals, one hundred and eight branch hospitals and dispensaries, scores of maternity and welfare centres, thirty leper settlements and clinics, a few tuberculosis sanatoria, and the famous medical and dental school of the West China Union University, the highest development in a varied and comprehensive system of training which is the backbone of our medical work; it is the recognition of a debt to the medical pioneers whose faith and vision laid the foundations so sure, and to their successors who took on from them the torch.

Much consolidation remains to be done before pressing challenges to advance can be met. The line is thin and its position precarious until it is further reinforced. Apart from recruiting, buildings and equipment in many places badly need renewal and additions. The present war is accentuating this problem, which was already serious before its outbreak.

Medical missions abroad constitute an advanced experiment in which every type of medical service has been linked on to the Church's life. Rich experience overseas is now available to all who would accept the responsibility for launching a campaign to bring the services of Medicine and of the Christian Church in evangelistic co-operation once again in this country. The way lies probably not through the intricacies of central planning and committee control, but through the study of local needs and the use of local personnel and resources in meeting them. Let us turn to the C.M.S. Medical Commission Report, 1939, for a summary of what an ideal Church medical policy should be.

Healing, we learn, must be taken to the sufferer ; to save involves seeking and not sitting ready in an institution waiting. Any adequate scheme must have an outreach into the homes of the people, where even in this island the great bulk of suffering and need is to be found. This involves a large personnel, and so all who recognize the call to such service must be mobilized to supplement those trained medical workers upon whom it has hitherto devolved. Personal relationships must supplement the work of clinics and hospitals. Healing demands suitable conditions in the home, and assistance often far beyond the facilities of most households. Concern and friendship are healing in themselves.

For such personnel, as for the more specialized, training is necessary. Hygiene, simple first aid, Christian psychology, should form its main features ; but the age, sex, and temperament of the helpers will determine what type of help each can best give. The need for help is great, and the forms it should take are multifarious. There are few experienced parish clergy, or general practitioners, or district nurses, who could not outline the special types of assistance for lack of which their work of ministering to suffering is handicapped. Many among them could also suggest or even organize the training locally of those willing to assist. As abroad, the opportunities for such lay-healing lie at our very doors !

Finally, the future of medical mission work abroad is increasingly to be found in preventive medicine. Most ill-health is preventable and could be prevented were the public convinced that the necessary precautions were worth the sacrifices involved. Knowledge of hygiene is more important than a first-aid certificate. Those in our own reception areas will realize how much remains to be done in this country—the pioneer in public health. With all its means of propaganda our Ministry of Health is still largely handicapped for lack of the active support of a friendly and concerned public opinion such as our churches could so well supply.

The Christian Church could do much if it were as convinced as the callowest newly-fledged doctor that disease is not inevitable. The wealth of ready generosity in its membership is shown by a study of the subscription lists of any charitable organization. Why should we remain content that Christian kindliness should be so largely dissipated in impersonal good deeds ? The home, the neighbourhood, the parish, these should be the spheres of service to which the Church again calls its members.

**The Future of Medical Practice.**

ON Sunday, 22nd March, Dr. Stark Murray, of the Socialist Medical Association, spoke to a meeting of members of the medical and ancilliary professions at the Victoria Rooms on "The Future of Medical Practice." The speaker, who is the editor of *Medicine To-day and To-morrow*, outlined in a most interesting way his ideas for the future organization of medicine in this country. He suggested that the basic unit would be the 1,000 bed hospital with a group of satellite health centres around, replacing the surgeries of the present-day practitioner. He stressed that the conception of team work in medicine was growing, that the suggested teams were becoming ever larger and more complex, and went on to make the point that the provision of such teams, their payment, and the provision of suitable operational bases would demand some form of unified and National Medical Service, financed directly from the national income. Such a system would, he claimed, ensure far better working conditions and facilities for doctors and those working with them, more post-graduate courses would be possible, and indeed would be compulsory ; the Government would have to make vastly increased provision for medical research.

Thus far the speaker had followed lines of thought familiar to all interested in the future of medicine in this country. He further pointed out that with the formation of the Nuffield Trust, the B.M.A. Planning Commission and the Ministry of Health's Inquiry, it seemed clear that the health services would probably be the next field for collectivistic experimentation, and that if the profession could put forward the broad outline of an agreed and comprehensive scheme, the more intimate details might be left to the profession to settle as it liked. But here, at the crux of the whole matter, the vital issue of control, the speaker became extremely vague. He denounced the half-and-half mentality, those who would agree with collectivization up to a point but could not face socialization. He castigated those who wanted to organize medicine on the lines of a Fascist corporation, but he did not even indicate how this was to be avoided if medicine is socialized in advance of other sections of the state. From his remark about the profession being allowed to work out the finer organizational details the speaker implied that, in the last analysis, the doctors would not be the masters in their own house. But who is to be in control ? He realized the widespread reluctance of the profession to trust its destiny to the very dubious mercies of the Ministry of Health, but seemed to think that a purged Ministry would be acceptable. But remembering that the speakers' journal had described the struggle of the New Zealand branch of the B.M.A. against the unfair conditions imposed by the Ministry of Health out there as " sabotage " (*Medicine To-day and To-morrow*, Sept., 1941. *Brit. Med. Journ.*, Nov. 22, 1941. Supplement), this proposition is not likely to be acceptable.

The main problem of the post-war world is going to be the fitting into the fabric of society of what may be broadly described as the " technicians." All hangs on this, particularly in medicine. What is to be the relationship of the profession to the public ? Who is to direct the health policies of the nation, those who know what they are doing, the medical scientists and technicians, are they to control or are they to trail along behind the slow-moving public opinion, always pinning their hopes to the education of the next generation, while ahead the advancing edge of scientific and technical achievement gets further out of sight ? Can there be a positive health policy unless there is restrictive or coercive legislation ? Is the medical profession to be coerced while the general public is permitted to flout the laws of health, the patent-medicine vendors flourish in the land and abuses of the healing art abound ? To all these questions Dr. Stark Murray gave no answer and no clear statement could be elicitated by questioning. Yet these are the vital matters into which we must inquire before committing ourselves to new ideas. The petty matters of local arrangements can soon be settled.

**Typhus in Britain.**

TYPHUS is so rarely seen in England and in recent years has not occurred in an epidemic form that the vivid account we publish in this issue, given by Dr. Kohn, who has been familiar with it in every form throughout his professional life, will be welcome to all our readers.

Those who heard the address delivered, without a note, in vigorous and fluent English will be glad to have this verbatim report. Greenwood, quoting figures up to 1931, was able to write that in the last two years, *i.e.* 1930 and 1931, no case of typhus had been notified in England and Wales, whilst in those two years Poland had 3,569 cases and U.S.S.R. 37,491. The last wide-spread epidemic in this country began in 1817 and continued in London through 1818 and 1819. According to Creighton almost every town and village of the United Kingdom was visited by typhus, although Christison said "all the great towns with the exception of Birmingham."

J. C. Prichard published in 1820 a *History of the Epidemic Fever which prevailed in Bristol*, 1817–19. During those years there were 591 cases in the poor's house (St. Peter's Hospital), 647 in the Royal Infirmary, 975 treated at home from the Dispensary, making 2,213 cases, of which public records were kept. But there were also many cases in private practice.

There had been at least one other well-authenticated earlier epidemic in Bristol, namely that referred to by Dr. Thomas Dover, in his *Ancient Physician's Legacy to his Country*, where he describes his work amongst the poor thus: "About fifty years since, this Fever raged much in Bristol so that I visited from twenty to thirty patients a day for a considerable time." This was the outbreak of

" spotted fever " which, in October, 1698, began to prevail all over England. Dover leaves no room for doubt that this spotted fever was indeed typhus, for he goes on to mention that he attended " near two hundred " poor children, " yet no more than one of them died of it of the whole number." An experience of typhus in children which is strictly according to rule.

The year 1698 was the worst of seven ill years at the end of the seventeenth century (1693–99), noted, says Thorold Rogers, " for the distress of the people and for the exalted profits of the farmer." Poverty gives typhus its opportunity to spread, not only by causing scarcity of food, but also by scarcity of soap ; for personal cleanliness and good laundry-work play an even more important part in eradicating typhus than does good feeding. Greenwood lays stress on " an adequate distribution and use of food, soap and water " for the control of typhus, which is " in theory the most controllable of diseases."

Creighton gives the year 1869 as being the time when typhus fever ceased to be epidemic or common, but on the later epidemics of fever he is an untrustworthy authority. For example, he includes the village epidemic at North Tawton (Devon) in 1839 as typhus, holding William Budd up to ridicule for maintaining that the disease was enteric and was spread by the excreta. Nowadays Budd's judgment is preferred to Creighton's.

Murchison regarded the epidemic of 1847 as being due to three different fevers, enteric, typhus and relapsing with a preponderance of typhus in Britain. He also states that in 1861 typhus again became epidemic, with the admission of 1,107 cases in the first six months of that year into the London Fever Hospital. After 1869 deaths from typhus, enteric and simple continued fever began to be tabulated separately in the Registrar-General's reports, although at the London Fever Hospital typhus and typhoid were recorded separately after 1849. Creighton's figures show a considerable prevalence of typhus in London in the years 1862–1868 ; based upon the London Fever Hospital admissions. It may be accepted that typhus has disappeared as an epidemic disease in Britain since 1869. But we must never forget that war may disturb the adequate distribution and use of food, soap and water : those three great safeguards of the public health.

REFERENCES.

Dover, T., *The Ancient Physician's Legacy to his Country*, 7th ed.

Greenwood, M., *Epidemics and Crowd-Diseases*. London, 1935.

Creighton, C., *A History of Epidemics in Britain*. Cambridge, 1894.

Prichard, J. C., *History of the Epidemic Fever, which prevailed in Bristol*, 1817–19. London, 1820.

Murchison, C., *Treatise on Continued Fevers*. London, 1862.

